# Reverse Genetics System for *Rabbit vesivirus*

**DOI:** 10.3389/fmicb.2020.596245

**Published:** 2020-11-13

**Authors:** Ángel L. Álvarez, Alberto García-Manso, Kevin P. Dalton, José M. Martín-Alonso, Inés Nicieza, Ana Podadera, Maikel Acosta-Zaldívar, Daniel de Llano, Francisco Parra

**Affiliations:** Departamento de Bioquímica y Biología Molecular, Instituto Universitario de Biotecnología de Asturias (IUBA), Universidad de Oviedo, Oviedo, Spain

**Keywords:** reverse genetics, calicivirus, infectious cDNA clone, virus rescue, *Vesivirus*

## Abstract

Most caliciviruses are refractory to replication in cell culture and only a few members of the family propagate *in vitro*. *Rabbit vesivirus* (RaV) is unique due to its ability to grow to high titers in several animal and human cell lines. This outstanding feature makes RaV an ideal candidate for reverse genetics studies, an invaluable tool to understand the molecular basis of virus replication, the biological functions of viral genes and their roles in pathogenesis. The recovery of viruses from a cDNA clone is a prerequisite for reverse genetics studies. In this work, we constructed a RaV infectious cDNA clone using a plasmid expression vector, under the control of bacteriophage T7 RNA-polymerase promoter. The transfection of permissive cells with this plasmid DNA in the presence of T7 RNA-polymerase, provided *in trans* by a helper recombinant poxvirus, led to *de novo* synthesis of RNA transcripts that emulated the viral genome. The RaV progeny virus produced the typical virus-induced cytopathic effect after several passages of cell culture supernatants. Similarly, infectious RaV was recovered when the transcription step was performed *in vitro*, prior to transfection, provided that a 5′-cap structure was added to the 5′ end of synthetic genome-length RNAs. In this work, we report an efficient and consistent RaV rescue system based on a cDNA transcription vector, as a tool to investigate calicivirus biology through reverse genetics.

## Introduction

**FIGURE 1:**
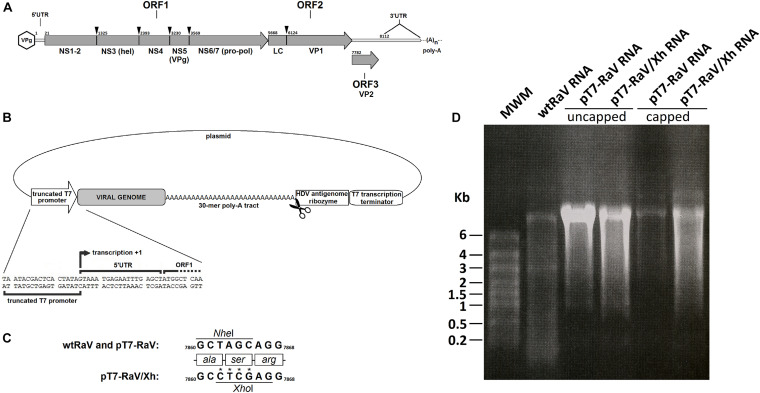
**(A)** Genomeorganization of *Rabbit vesivirus*. The proteolytic cleavage of ORF1-encoded polyprotein, as well as the capsid leader (LC) cleavage site, are indicated with arrowheads, along with the names of mature peptides generated. Numbers refer to nucleotide positions within the genome. **(B)** Schematic representation of the genetic elements in the infectious clone design, including the nucleotide sequence neighboring the +1 transcription site. Scissors represent the HDV antigenome ribozyme cleavage site. **(C)** Detail of the site-directed mutagenesis of nucleotide residues 7,862–7,865 for the generation of tagged RaV, involving the loss of a *Nhe*I restriction site and the appearance of a novel *Xho*I site. Numbers refer to nucleotide positions within the genome. **(D)** Formaldehyde-agarose denaturing electrophoresis gels. Analysis of capped (+) and uncapped (–) genome-length RaV RNA products, obtained using *in vitro* transcription of *Not*I-linearized pT7-RaV and pT7-RaV/Xh infectious clones. Virion RNA extracted from a concentrated purified RaV stock was run in the first lane, for comparative purposes.

*Rabbit vesivirus* (RaV) was first isolated in the Veterinary Diagnostic Laboratory at Oregon State University, from feces of rabbits suffering gastrointestinal disorders. The virus was characterized, and a cDNA copy of the genome was cloned and sequenced. The virus was found to be non-enveloped, isometric and around 30 nm in diameter. The viral genome is 8,295 nucleotides (nt) in size and consists of positive-sense single-stranded RNA with a small protein (VPg) covalently linked to its 5′ terminus and a 3′ poly-A tail of an average length of 85 nt. The genome comprises 3 open reading frames (ORFs) the most 5′ of which encodes a 1,880 amino acids polyprotein that yields the non-structural polypeptides upon self-cleavage. The comparative analyses of domain homology and proteolytic cleavage sites with respect to other caliciviruses and picornaviruses allowed the establishment of the number of mature non-structural proteins, their putative functions, and a hypothetical processing map (Figure 1A). RaV ORF1-encoded non-structural proteins include NS1-2 precursor (unknown function), NS3 (NTPase, helicase), NS4 (membrane rearrangement during replication), NS5 (genome-linked viral protein, VPg), and NS6/7 (a bi-functional mature polypeptide containing both, the cysteine-protease activity responsible for polyprotein self-processing and the RNA-dependent RNA polymerase or viral replicase). Based on these features and other serological and phylogenetic criteria, it was classified among the *Caliciviridae*, within the *Vesivirus* genus (Martín-Alonso et al., 2005).

While most caliciviruses are unable to grow in cell cultures, the *Vesivirus* is the only genus in which all its members are cultivable (Clarke and Lambden, 1997). Most vesiviruses show a tight host-specificity restriction for *in vitro* replication, only infecting cell lines derived from their animal hosts (e.g., *Feline calicivirus*) (Pesavento et al., 2008), whereas a few may cross the species barrier and infect new animal hosts, even from a distinct habitat (e.g., *San Miguel sea lion virus*/*Vesicular exanthema of swine virus*) (Neill et al., 1995) the latter being more likely to infect a broader spectrum of cell cultures from different species. In fact, though it was initially isolated from deceased rabbits, we consider RaV to be a member of a subgroup known as “marine” or “ocean-originated caliciviruses,” a well-established clade within the caliciviruses, thought to have reached terrestrial species through feedings made from marine organisms (Martín-Alonso et al., 2005). Because of its genetic proximity to the etiological agent of vesicular exanthema of swine, a communicable eradicated disease, a moratorium on animal experimentation with RaV has been observed. Thus, our knowledge regarding this virus tropism, host range and pathogenesis is rather scarce and limited to data collected from the rabbits from which it was first isolated (Martín-Alonso et al., 2005).

A relevant feature of RaV is its ability to efficiently replicate to high titers in a wide range of mammalian (including human) cell lines, such as Vero, RK13, 293T, HeLa, HeLa-S3, among others. This ability makes this virus an ideal candidate for a reverse genetics system that represents an invaluable tool for understanding the molecular basis of virus replication and the biological functions of viral genes. Reverse genetics also opens new avenues for vaccine development based on attenuated viruses, the use of recombinant replicons or viruses as vectors for heterologous gene expression with potential biotechnological applications and the screening of novel compounds with potential antiviral effects.

Due to the technical difficulties to modify RNA, reverse genetics studies with RNA viruses require that the nucleotide changes be made on a cloned cDNA copy of the genome, obtained by reverse transcription. The rescue of infectious virions from such vectors is a prerequisite for any kind of reverse genetics study (Boyer and Haenni, 1994; Goodfellow, 2013). The first rescue of a calicivirus took place after transfection of cat kidney-derived CRFK cells with RNA obtained by *in vitro* transcription from a *Feline calicivirus* (FCV, genus *Vesivirus*) genome-length cDNA clone. This clone was assembled through restriction endonuclease digestions and subsequent ligations of several fragments covering the whole viral genome. The cDNA was linearized prior to transcription to obtain transcripts with a defined 3′ end (run-off), using the T7 phage polymerase in the presence of high concentrations of a cap analog (Sosnovtsev and Green, 1995). A more elegant version of the FCV reverse genetics system was obtained later, in which a recombinant helper virus was used to provide the T7 phage RNA polymerase *in trans* (MVA-T7), allowing the cDNA to be transfected directly, bypassing the *in vitro* transcription step (Mitra et al., 2004).

Following the initial success achieved with FCV, the systematic application of this methodology has led to the establishment of several reverse genetics systems for other caliciviruses, such as *Porcine enteric calicivirus* (PEC) (genus *Sapovirus*) which is the only cultivable sapovirus (Chang et al., 2005) and *Murine norovirus* (MNV, genus *Norovirus*) (Chaudhry et al., 2007; Yunus et al., 2010).

From the very first description of RaV in our laboratory, the establishment of a reverse genetics system has been one of our principal objectives. In this work, we demonstrate the consistent and reproducible recovery of RaV infectious progenies after transfecting cells either with plasmids encoding the full-length genomic cDNA under the control of T7 phage RNA-polymerase promoter, or from synthetic genome-length RNA transcripts generated from such plasmids by *in vitro* transcription.

## Materials and Methods

### General Experimental Procedures

Ultra-pure water used in molecular biology applications was obtained with a deionizer Milli-Q Plus equipment (Merck, Millipore, Germany). Chemical reagents including organic solvents and inorganic compounds were from Merck, GPR, and BDH. Protein electrophoresis materials and reagents were from Bio-Rad (China) while nucleic acids electrophoresis materials were from Sigma-Aldrich. Cell culture mediums and materials were from Lonza, Gibco, Corning and BD Biosciences. PCR reactions were carried out using the Long and Accurate (LA) Taq PCR kit (Fermentas). The oligonucleotides used as primers for PCR throughout this work are listed in Table 1. Molecular weight ladders used in this study were the O’Generuler 1 Kb Plus DNA ladder (Fermentas) for DNA electrophoresis as well as PageRuler^TM^ NIR prestained protein ladder (Fermentas) for protein electrophoresis. All procedures involving RaV were performed in a biosafety class 2B1 cabinet, following the appropriate biological security guidelines.

**TABLE 1:** Oligonucleotides.

***Name***	***Sequence (5***′***→3′)***
*Ase*I-T7-5′RaV (fw)	CATGCATGCGATTAATGGTAATACGACTCACTATAGTAAATGAGAATTTGAGCTATGGCTCAAACGCTCTCG
RaV_Nh_Xh (fw)	GGAAAGCGGATTGACCTCGCCTCGAGGAATCTGGCTCTC AAAAATCGC
RaV_Nh_Xh (rv)	GCGATTTTTGAGAGCCAGATTCCTCGAGGCGAGGTCAATC CGCTTTCC
RaV2	AACTAGTCCGTTTTGTAGAAGC
RaV-2ABC (rv)	ATCACGTTGTCAAGTGCAGACATCAG
RaV32	ATTCTAACAACAAATTGGAACCAAG
RaV-5′-*Sal*I (fw)	GATCGATCGAGTCGACGTAAATGAGAATTTGAGCTATGGC
RaV-*Age*I (fw)	GATTCGACTACAACCGGTTGGTC
RaV-*Age*I (rv)	GACCAACCGGTTGTAGTCGAATC
RaV-OL-A30 (fw)	CATTAGGAAAAAAAAAAAAAAAAAAAAAAAAAAAAAAGGG TCGGCATGGCATCTCCAC
RaV-OL-A30 (rv)	GTGGAGATGCCATGCCGACCCTTTTTTTTTTTTTTTTTTT TTTTTTTTTTTCCTAATG
RaV-RACE-GSP1 (rv)	CGTAGAGTGCGCAACTAGG
T7Ter-*Not*I (rv)	CGATCGATGCGGCCGCGATCTCGATCCGGAT ATAGTTCCTC
IC-linker-Fw	GGCCGCTAACCATATGTCGAGAGCTACGTAGTAGCG
IC-linker-Rv	TCGACGCTACTACGTAGCTCTCGACATATGGTTAGC

### Viruses and Cells

*Rabbit vesivirus* (RaV) was kindly provided by Prof. Alvin Smith (Veterinary Diagnostic Laboratory at Oregon State University). RaV was initially isolated from a rabbit showing gut pathology, liver damage and diarrhea. The virus was adapted to cell culture and characterized in our lab, its genome was cloned and sequenced and the prototype RaV sequence was deposited in GenBank (accession number: AJ866991) (Martín-Alonso et al., 2005).

Vero cells (ECACC, 84113001) were used for the generation of RaV stocks and their titration by both the plaque assay and the Reed and Muench end-point dilution method (Reed and Muench, 1938). 293T cells (ATCC, CRL-11268) were used for transfection of plasmids and synthetic viral RNA during the virus recovery assays. Both cell lines were grown in Dulbecco-modified Eagle’s minimal essential medium (DMEM) supplemented with 10% bovine fetal serum and 50 μg/mL gentamicin at 37°C in a 5% CO_2_ atmosphere and were maintained in the same medium with the serum concentration reduced to 2%.

A recombinant strain of *Fowlpox virus* expressing the bacteriophage T7 RNA-polymerase (rFPV-T7) was kindly donated by Professor Paul Britton (Animal Health Institute, Compton, United Kingdom) and was propagated and titrated using primary cell cultures from chicken embryo fibroblasts (Britton et al., 1996).

### RaV Expression Constructs

Total RNA extracted from RaV-infected Vero cells was used to obtain the viral full-length genome, by reverse transcription using oligo-dT_20_ for the synthesis of the first DNA-strand. Two contiguous fragments of the viral genome were cloned individually into pGEM-T Easy vector (Promega) before their final assembly. A large genome fragment (6,927 bp) was obtained using the oligonucleotides RaV-5′-*Sal*I (fw) and RaV-*Age*I (rv). This amplicon contained a *Sal*I restriction site in its 5′ terminus (upstream the virus genome start) and included a unique viral *Age*I site (ORF2) in its 3′ terminus. This amplicon was used to produce pGEM-5′RaV/*Sal*I-*Age*I vector (9,944 bp). A second fragment (1,443 bp) was amplified with oligonucleotides RaV-*Age*I (fw) and RaV-OL (rv). This fragment was fused, using overlapping PCR, to a regulatory cassette containing the sequence of *Hepatitis delta virus* (HDV) antigenomic ribozyme followed by the T7 promoter terminator signal and a *Not*I restriction site. The regulatory cassette was cloned from the Andrew Ball’s transcription vector 2.0 (Pattnaik et al., 1992) using primers RaV-OL (fw) and T7Ter-*Not*I (rv). The resulting construct pGEM-3′RaV/*Age*I-*Not*I (4,687 bp) contained the RaV genome 3′ end including the 3′-UTR, a 30-mer poly-A tract, the ribozyme and the transcription terminator cloned into pGEM-T Easy. This plasmid DNA was cut with *Sal*I and *Age*I enzymes and served as the acceptor of the large *Sal*I/*Age*I fragment from pGEM-5′RaV/*Sal*I-*Age*I, that completed the RaV full-length genome sequence. Because of the presence of SP6 phage promoter, the resulting vector was named pSP6-RaV, though it was not adequate for expression, but used for sequencing. In addition, this vector was the primary source of RaV genome sequence for further sub-cloning and generation of genome-expressing vectors.

The pSP6-RaV plasmid was used as the template for PCR-amplification of a 5′ genomic fragment using the primers *Ase*I-T7-5′RaV (fw) and RaV-2ABC (rv). This fragment contained a truncated version of T7 RNA-polymerase promoter, lacking two 3′-terminal G nucleotides to allow the correct positioning of the first viral residue in the transcription +1 site. The amplicon was inserted into the cloning vector pJET1.2/blunt (Fermentas), leading to pJET-T7-T7RaV (4,728 bp). The correct orientation was checked by sequencing and a clone in which the native T7 promoter present in the cloning vector was flanked by *Not*I and *Pst*I sites was selected. A *Not*I-*Pst*I spacer from an unrelated sequence was introduced in order to delete the redundant T7 promoter. The resulting plasmid was digested with *Hpa*I and *Not*I and the vector backbone served as the acceptor for the *Hpa*I-*Not*I fragment from pSP6-RaV. This ligation reaction completed the full-length RaV genomic sequence under the control of a truncated T7 promoter, together with the 3′ elements 30-mer poly-A tract, HDV ribozyme and T7 terminator signal. Finally, the construct was digested with *Sal*I and *Not*I to replace the 759 bp-spacer by a short compatible linker obtained through annealing of the complementary oligonucleotides IC-linker-Fw and IC-linker-Rv. The final plasmid named pT7-RaV (11,247 bp) was used as infectious clone to recover RaV progenies from cell cultures.

For differential identification of RaV recovered from pT7-RaV, four nucleotides were substituted by site-directed mutagenesis, using the Quickchange II XL mutagenesis kit (Stratagene). The mutagenic primers used were RaV_Nh_Xh (fw) and RaV_Nh_Xh (rv). The resulting infectious clone pT7-RaV/Xh (11,247 bp) differs from the wild type RaV genome in the substitution of a ubiquitous *Nhe*I restriction site by a novel *Xho*I site within the coding sequence of VP2 protein with no amino acid change.

### *In vitro* Transcription and Capping of Synthetic Viral RNA

The pT7-RaV and pT7-RaV/Xh constructs were linearized with *Not*I and used in *in vitro* transcription assays to produce synthetic genome-length viral RNA, using the RiboMAX Large scale T7 RNA production system (Promega), following the manufacturer specifications. The synthetic RNA was extensively treated with RNase-free DNase (Qiagen) to remove the DNA template. The 5′-cap structure was post-transcriptionally added to synthetic transcripts with the aid of Scriptcap system (Epicenter) following the manufacturer specifications and the capped RNA was cleaned-up using the RNeasy kit (Qiagen).

### Formaldehyde-Agarose Denaturing Gel RNA Electrophoresis

The integrity and size of both, *in vitro* transcribed and virion-purified RNA were checked through electrophoretic separation on 1% agarose gels under denaturing conditions (6.7% formaldehyde) in MOPS buffer (5 mM sodium acetate, 1 mM EDTA, 20 mM MOPS). Briefly, 2–3 μL of uncapped *in vitro* transcribed RNA, 3–5 μL of capped *in vitro* transcribed RNA, or 1–3 μL of RaV genomic RNA purified from virions were mixed with identical volume of 2X RNA sample loading buffer (Fermentas). The samples were heated to 65°C for 10 min, immediately cooled on ice for 2 min and loaded onto the gel and ran at 5V/cm for 30 min. Ethidium bromide was present in the sample loading buffer and RNA was visualized under UV. The high-range (HR) RiboRuler ladder (Fermentas) was used to estimate RNA approximate sizes.

### Recovery and Passage of Infectious RaV

Twelve-well plates seeded with 293T cells grown to 70% confluence were transfected with 1 μg/well of pT7-RaV- or pT7-RaV/Xh-derived, capped or uncapped, synthetic RNAs obtained in *in vitro* transcription reactions. For each well, 4 μL of lipofectamine 2000 (Invitrogen) were used according to the manufacturer’s recommendation.

The procedure used for DNA transfections was similar to that of RNA transfection, described above. Briefly, 293T cells were grown to approximately 70% confluence in 12-well plates for 24 h then the growth medium was removed, and the wells were filled with maintenance medium. Each well was then infected with rFPV-T7 (MOI = 10) and the plates were incubated for 2 h at 37°C to allow virus adsorption and entry. DNA transfection mixes were prepared using 4 μL/well of lipofectamine 2000 (Invitrogen) and 1.6 μg of either pT7-RaV, pT7-RaV/Xh or the control plasmid pT7-GFP) according to the manufacturer’s protocol. After infection with rFPV-T7 the inocula were removed, 500 μL of fresh OPTI-MEM were added to each well, and the transfection mixes were finally added to the well dropwise. The plates were gently rocked and incubated for 24–48 h at 37°C.

After incubation, the cells transfected with either synthetic RNA or with plasmid DNA in the presence of rFPV-T7 infection (passage 0), were scrapped within the transfection medium using a pipette tip, collected and pelleted by centrifugation at 1,000 *g* for 10 min. The supernatants were used to inoculate fresh cultures of confluent Vero cells (passage 1). All supernatants from rFPV-T7 infected samples were filtered through 0.2 μM membranes (Millipore) prior to their inoculation in Vero cells to remove or minimize the poxvirus in subsequent inoculations. The pelleted cells were used for protein (Western blot) or RNA analysis (RT-PCR). The procedure described above was repeated to obtain cells and supernatant samples from up to 5 blind passages of rescued viruses. For convenience, viruses recovered from cells transfected with pT7-RaV or pT7-RaV-derived RNA were named rRaV (for rescued RaV) while viruses rescued from pT7-RaV/Xh or pT7-RaV/Xh-derived RNA were named rRaV/Xh (accounting for rescued RaV with *Xho*I tag). Stocks of these viruses were made by inoculating T150 flasks containing confluent Vero cell monolayers with the corresponding supernatants from passages 3. Concentration and purification of virus were performed using a combination of two ultracentrifugation rounds and an intermediate step of freon extraction, as described previously (Martín-Alonso et al., 2005).

### Characterization of Rescued RaV Progenies

Virus stocks were used for RNA extraction, RT-PCR and cloning for automatic sequencing purposes. The nucleotide sequences obtained were compared to that of prototype RaV available in GenBank (AJ866991). Additionally, the sequences of 5′-ends of viral RNA from rescued viruses were investigated using the system for rapid amplification of 5′ cDNA ends (5′-RACE) (Invitrogen) according to manufacturer’s instructions. Briefly, reverse transcription was performed using RaV-RACE-GSP1 (rv), an antisense primer annealing close to the 5′-end, and the Superscript II reverse transcriptase enzyme provided with the kit. The RNA template was removed with RNase H and a poly-dC tail was added to the 5′-end of single stranded cDNA, using the terminal deoxynucleotidyl transferase (TdT) in the presence of dCTP. The abridged anchor (fw) primer provided (Invitrogen) containing a 3′-terminal poly-dG tract, together with RaV18 primer were used for the amplification of RaV 5′-end. The amplicons (approx. 488 bp) were cloned and sequenced.

RT-PCR reactions were also carried out using total RNA samples extracted from Vero cells after 36 h of infection with rRaV and rRaV/Xh (passage 3) at MOI = 0.1, using Superscript III reverse transcriptase kit (Invitrogen). The oligonucleotides used for PCR were RaV32 and RaV2, which spanned a region within VP2 coding sequence including the molecular tag *Xho*I. The RNA was extensively treated with 50 Kunitz units of RNase-free DNase I (Qiagen) for 1 h at 37°C and cleaned-up (RNeasy, Qiagen) prior to RT. A negative control without RT enzyme was always set along with each sample (to ensure that amplicons come from RNA). The PCR amplicons were analyzed for *Xho*I restriction site by digestion with this endonuclease.

293T cells transfected with either synthetic viral RNA or infected with rFPV-T7 and transfected with pT7-RaV or pT7-RaV/Xh, as well as Vero cells from subsequent passages 1 to 3 of these transfections, were scrapped at 36 hpi and suspended in protein sample buffer for SDS-PAGE. The protein samples (5 μL each) were analyzed by SDS-PAGE in 10% polyacrylamide gels run at 150 mA for 1 h in a Mini-PROTEAN 3 apparatus (Bio-Rad). A Semi-Phor TE70 apparatus (Hoefer Scientific Instruments) was used to electrotransfer proteins to Immobilon FL membranes (Millipore) at 150 mA for 45 min in transfer buffer (0.248 M Tris–HCl pH 8.8, 1.92 M glycine, 20% methanol). The membranes were blocked by rocking for 1 h in PBS containing 5% skimmed milk and further incubated for 1 h with a 5% skimmed milk and 0.05% Tween 20-PBS solution containing the primary antibody. After 5 min washing with 0.05% Tween 20-PBS solution, the membranes were incubated for 1 h at room temperature with the secondary antibody. The blots were then washed twice with PBS for 30 min and the reactive protein bands were visualized by fluorescence using an Odyssey Infrared Imaging System (LI-COR Biosciences). The primary antibodies used throughout this study were rabbit polyclonal antisera raised against RaV NS3 and VP1 proteins diluted 1:500 and 1:2,000, respectively, as well as commercial monoclonal antibodies against tubulin (DM1A mAb, Calbiochem, cat. No. CP06), diluted 1:1,000. The secondary antibodies used were either IRDye 800CW goat anti-rabbit IgG H + L or IRDye 800CW goat anti-mouse IgG H + L, both purchased from LI-COR BIOSCIENCES, diluted 1:20,000 in 5% skimmed milk, 0.05% Tween 20-PBS.

One-step growth curves for wtRaV and rRaV/Xh were obtained. Briefly, confluent Vero cell monolayers grown in 12-well plates were pre-incubated 1 h at 4°C and inoculated with rRaV/Xh or wtRaV at MOI = 10. After 1 h-adsorption at 4°C the inoculum was removed, and the cells were washed with ice-cold PBS. Fresh maintenance medium was added, and the temperature was raised to 37°C to allow virus entry. Samples from supernatants and cells were collected separately at 2, 3, 4, 6, 8, and 14 hpi. The supernatants were titrated in Vero cells by plaque assay and the cell pellets were analyzed by Western blot for viral protein detection, as described above.

The effect of transfecting DNA amount in the rFPV-T7 system over the titer of rescued viruses was evaluated through plaque assay. Briefly, 70% confluent 293T cell monolayer were infected with rFPV-T7 as previously described, and further transfected with either 1, 3, or 5 μg of pT7-RaV/Xh. After 24 h, the supernatants from transfections (1 mL) were used to inoculate fresh Vero cell monolayers (passage 1). 24 h later, the supernatant from passage 1 were titrated by plaque assay, as previously described (Chaudhry et al., 2007).

### Bioinformatics and Statistics

The nucleotide sequences data collected from the automatic sequencing service were processed with Chromas v2.31 (Technelysium Pty Ltd). The Vector NTI 8 Suite (InforMax Inc.) was used to design PCR primers, cloning strategies, to predict restriction digestions results and for alignments, assembly of contiguous fragments and *in silico* translations of nucleotide sequences.

The SPSS Statistics software v22 (IBM) was used to analyze the statistical significance of differences found among wtRaV and rRaV/Xh titers during one-step growth. The Kolmogorov-Smirnov and Levene tests were employed to assess normality and homoscedasticity, respectively (*p* > 0.05). Student’s *t*-tests were used to compare wtRaV and rRaV/Xh titers within each time point.

## Results

### *Rabbit vesivirus* Infectious Clones

The main goal of this work was to construct a DNA vector capable of directing the synthesis of the *Rabbit vesivirus* genomic length RNA, the translation of which would lead to replication and give rise to a progeny of infectious virions. To efficiently generate a viable virus progeny, an infectious clone must contain a cDNA of the full virus genomic sequence properly placed under the control of a suitable RNA polymerase promoter to direct the transcription of the genetic construct within the cell or *in vitro* (Boyer and Haenni, 1994).

In the case of RaV, as for the rest of caliciviruses, the first event occurring after genome uncoating in the cytoplasm, is ORF1 translation. *Vesivirus* ORF1 encodes a multifunctional polyprotein co-translationally cleaved into mature viral non-structural polypeptides NS1 to NS6/7 (Figure 1A). NS6/7 is a bi-functional protease-polymerase that copies the viral genome producing a full-length complementary (negative) strand. The negative strand serves as a template for the synthesis of both, the full-length positive (genomic) RNA and a smaller RNA species of about one-third of the genome length (subgenomic RNA, sgRNA), encoding ORF2 and ORF3 (major and minor virion structural proteins, respectively) (Green et al., 2000). The genomic organization of RaV is depicted in Figure 1A.

When producing genome-length RNA from expression vectors, the addition of non-viral sequences upstream of the full-length RNA transcript, generally compromises virus viability and recovery (Bridgen and Elliot, 2000). To avoid this, the RaV genome-expressing vector pT7-RaV was engineered to contain a truncated version of T7 phage RNA polymerase promoter in which the transcription start (+1 site) coincides with the first RaV genome nucleotide (Figure 1B). The occurrence of an authentic viral genome 3′-end can also be essential for virus replication. Thus, pT7-RaV construct incorporates an HDV antigenomic ribozyme sequence immediately downstream of the poly-A tract. This autocatalytic RNA sequence folds back and cuts, separating itself from the poly-A tract releasing a 30-mer poly-A tail. Additionally, a T7 transcription terminator placed downstream the HDV ribozyme sequence will ensure length homogeneity of RNA transcripts and proper functioning of the ribozyme (Figure 1B). This vector was checked by automated Sanger sequencing and comparison with the prototype RaV reference sequence, previously deposited in GenBank (accession number: AJ866991).

A modified version of pT7-RaV vector was constructed (namely pT7-RaV/Xh), using site-directed mutagenesis, to incorporate a molecular tag that allows recombinant virus rescued upon plasmid delivery, to be distinguished from a casuistic wild type RaV contamination. This molecular tag consisted of four nucleotide changes that led to the suppression of a *Nhe*I restriction site and the appearance of a novel unique *Xho*I site within the VP2 (minor capsid protein) coding sequence, without altering the protein primary structure (Figure 1C). The construct pT7-RaV/Xh was sequenced using the automated Sanger system and the resulting nucleotide sequence was deposited in GenBank and assigned the accession number MN179279. The pT7-RaV and pT7-RaV/Xh constructs generate, in the presence of T7 RNA polymerase, genome-length transcripts of a size similar to that of wtRaV genomic RNA (Figure 1D).

### pT7-RaV and Its Tagged Version pT7-RaV/Xh Can Produce Infectious RNA

**FIGURE 2:**
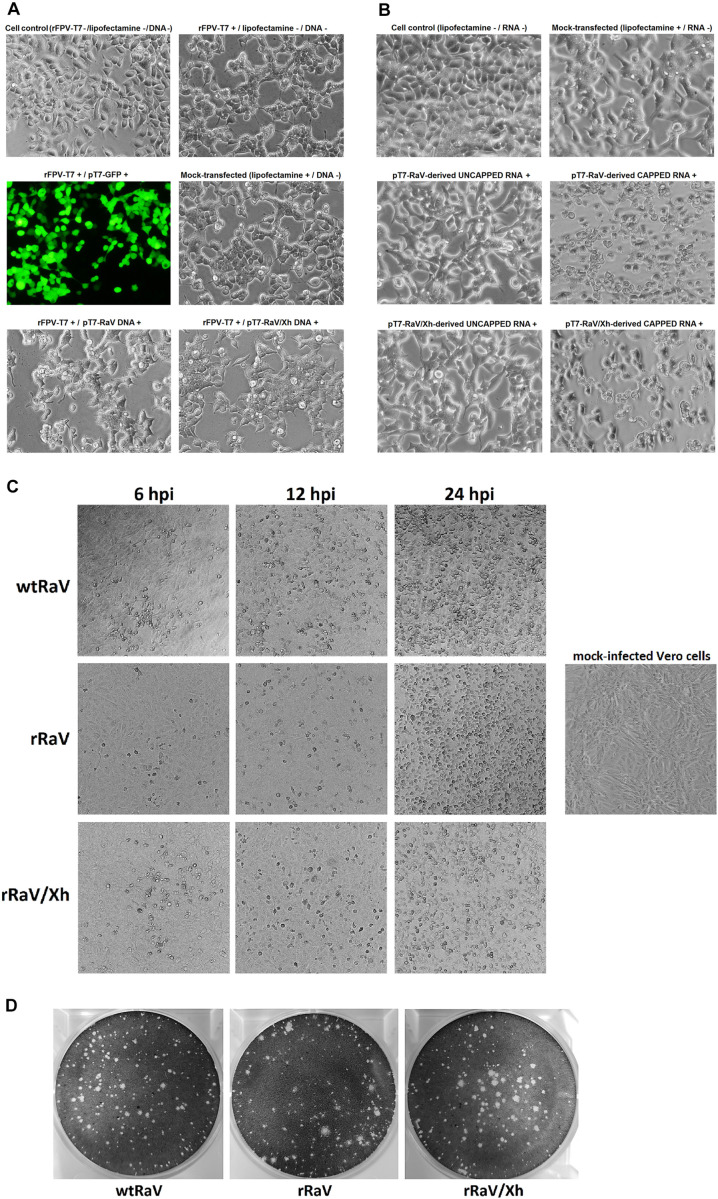
Microscope images (100 X) of 293T cells either **(A)** infected with rFPV-T7 and further transfected with DNA infectious clones or **(B)** transfected with capped or uncapped synthetic genome-length viral RNA. GFP expression from pT7-GFP plasmid visualized using a fluorescence filter served as a transfection efficiency control. **(C)** Bright field microscope images (40 X) of Vero cells infected with either wtRaV, rRaV, or rRaV/Xh (MOI = 0.1, passage 3). The photos were taken at 6, 12, and 24 hpi. **(D)** Morphology of wild-type and rescued RaV lysis plaques in Vero cells under 1% agarose overlay.

293T cells infected with rFPV-T7 and transfected with pT7-RaV or pT7-RaV/Xh plasmids appeared damaged probably because of FPV-induced cytopathic effect (CPE) itself, preventing the RaV-induced CPE from being clearly observed in passage 0 (Figure 2A). Conversely, cells transfected with RNA transcripts (derived from pT7-RaV or pT7-RaV/Xh) showed a different pattern: in transfections with uncapped RNA, the cell monolayers were indistinguishable from those of lipofectamine-treated mock-transfected cells; transfection of capped synthetic RNAs derived from pT7-RaV or pT7-RaV/Xh led to a CPE clearly distinct from that observed in lipofectamine treated mock-transfected and uncapped RNA-transfected cells. This CPE consisted of cell rounding and refringency, reminiscent of virus-induced CPE (Figure 2B).

The supernatants from cells transfected with either pT7-RaV DNA, pT7-RaV/Xh DNA in the presence of rFPV-T7 infection, or their capped derived RNA transcripts synthesized *in vitro* were infectious when inoculated into fresh Vero cell monolayers (passage 1) and typical virus-induced CPE subsequently appeared after each serial supernatant passage. Figure 2C shows Vero cell monolayers infected with virus stocks (MOI = 0.1) generated from either wtRaV, rRaV recovered from rFPV-T7/pT7-RaV DNA transfection or rRaV/Xh recovered from rFPV-T7/pT7-RaV/Xh DNA transfections, after three virus passages. The CPEs recorded for rescued viruses rRaV and rRaV/Xh were similar to that induced by wtRaV and also showed comparable time progression. The CPE consisted of cell rounding, refringency and a progressive loss of cell confluence as the viruses spread and infect the whole monolayer. An identical effect was observed during infections with stocks from viruses recovered after transfections with *in vitro* synthesized genome-length capped RNAs (data not shown).

A plaque assay was also performed to compare the morphology of virus plaques. rRaV and rRaV/Xh recovered from rFPV-T7/DNA transfections (as well as those recovered after RNA transfections, data not shown) developed lytic plaques similar to those of wtRaV (Figure 2D).

**FIGURE 3:**
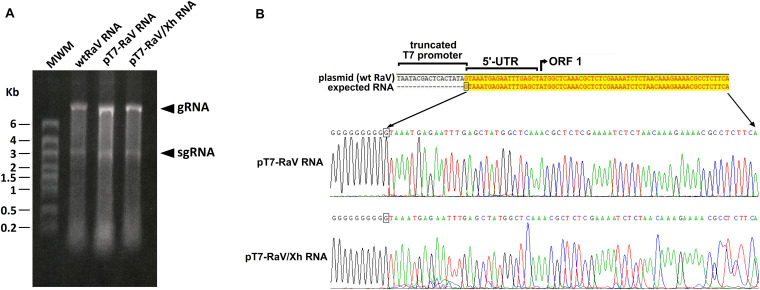
**(A)** Formaldehyde-agarose denaturing gel analysis of virion RNA extracted from concentrated stocks of wtRaV, rRaV, and rRaV/Xh. gRNA: genomic RNA; sgRNA: subgenomic RNA; MWM, molecular weight marker High Range (HR) RiboRuler (Fermentas); Kb, kilobases. **(B)** Sequences of RNA 5′ ends found after 5′-RACE assays. RNA was extracted from cells infected with rFPV-T7 and further transfected with pT7-RaV or pT7-RaV/Xh (passage 0). The first 430 nt of the 5′ ends of pT7-RaV and pT7-RaV/Xh-derived transcripts were identical to those of wtRaV RNA, though only the first 67 nt are shown.

We also studied the integrity of the RNA packaged within the virions of rescued RaV compared to wild type. As shown in Figure 3A, the RNAs extracted from concentrated stocks of rescued viruses rRaV and rRaV/Xh were similar in size to those present in wtRaV virions, and consisted of two RNA species: the full-length genomic RNA (gRNA, ≈8.3 Kb) and the subgenomic RNA (sgRNA, ≈2.6 Kb).

The integrity of 5′ ends of pT7-RaV and pT7-RaV/Xh-derived RNA transcripts generated in transfected cells (extracted from passage 0 cells), was checked through a 5′-RACE assay. All 5′ ends sequences found for RNA transcripts derived from the RaV infectious clones were confirmed to be identical to that of RNA transcripts produced during wtRaV infection (Figure 3B).

**FIGURE 4:**
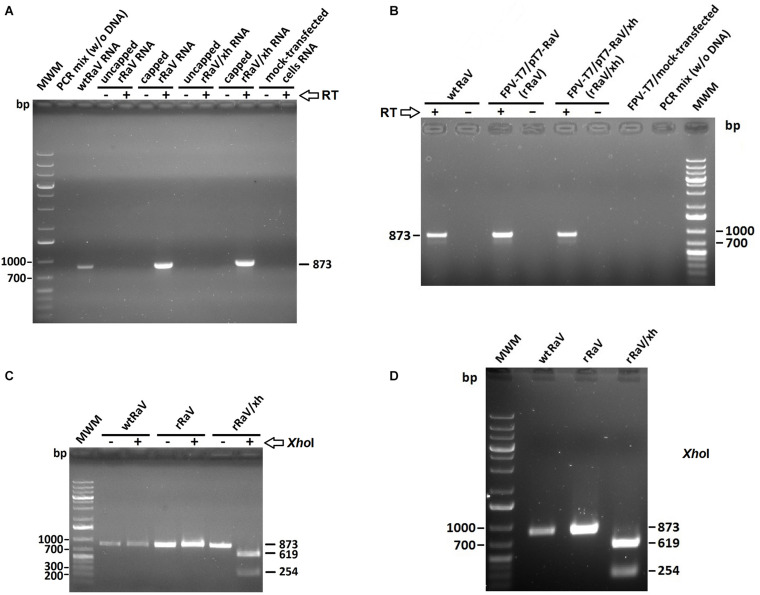
Agarose gel electrophoresis of RT-PCR products derived from *de novo* synthesized viral RNA in cells infected with rescued viruses rRaV and rRaV/Xh after 2 blind virus passages. **(A)** Amplicons from cells infected with RaV (passage 3) recovered after transfection using synthetic capped or uncapped genome-length RNA transfections (capped or uncapped refers to RNA used in passage 0). The absence or presence of RT enzyme during the first strand synthesis step is indicated by –/+, respectively. **(B)** Cells infected with RaV recovered after rFPV-T7 infection followed by DNA transfection. The absence or presence of RT enzyme during the first strand synthesis step is represented by –/+, respectively. **(C,D)** Digestion products obtained after treating the PCR amplicons from A and B with *Xho*I restriction enzyme. The –/+ signs indicate the absence or presence of *Xho*I enzyme when applicable. MWM, molecular weight marker O’Generuler 1 Kb Plus DNA ladder (Fermentas); bp, base pairs.

We assessed the presence of *de novo* synthesized viral RNA through RT-PCR from cells infected with rescued rRaV and rRaV/Xh viruses (passage 3). For virus rescue assays using transfection of *in vitro* synthesized genome-length RNA transcripts, we additionally evaluated the role of 5′-cap structure in virus recovery. As can be seen in Figure 4A, the expected PCR products (873 bp) were only obtained from *in vitro* synthesized RNA transcripts subjected to post-transcriptional capping prior to transfection. Transfection of 293T cells (passage 0) with either pT7-RaV or pT7-RaV/Xh-derived *in vitro* generated capped RNAs yielded viral RNA beyond three supernatant passages in Vero cells (passage 3). For cells transfected with plasmid DNA (pT7-RaV and pT7-RaV/Xh) an extensive DNase treatment was carried out prior to RT-PCR and control reactions without RT enzyme were set up in parallel, to rule out DNA template amplification. As shown in Figure 4B, the expected PCR products (873 bp, from the region comprising nt 7,244–8,116) were only obtained if RT enzyme was present during the first strand synthesis step, suggesting that the observed amplicons are derived from *de novo* synthesized viral RNA and not from remaining plasmid DNA used for transfection.

As previously explained, we introduced a novel unique *Xho*I restriction site into the infectious clone pT7-RaV that led to the pT7-RaV/Xh infectious construct (Figure 1C). Then, we investigated the presence or absence of this molecular tag within rescued virus RNA isolated from infected Vero cells (passage 3). For this purpose, the 873 bp above-mentioned amplicons (that covered the region where the tag was inserted) were digested with *Xho*I enzyme. As can be seen, only amplicons derived from rRaV/Xh-infected cells were positive for *Xho*I cut, yielding two smaller fragments of 619 and 254 bp (Figures 4C,D).

### Rescued and Wild-Type RaV Display Similar Viral Protein Expression and Growth Profiles

**FIGURE 5:**
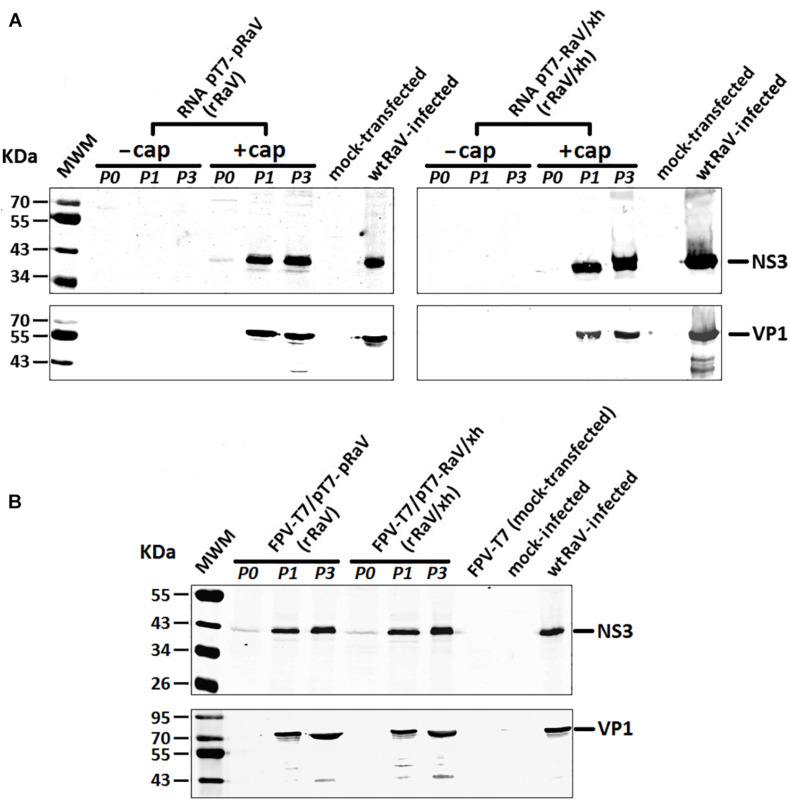
Western blot analyses of RaV NS3 and VP1 proteins during passages 0, 1, and 3 of rescued rRaV and rRaV/Xh using either **(A)** synthetic capped or uncapped RNA transfections, or **(B)** rFPV-T7 infection followed by DNA transfections. MWM, molecular weight marker Broad Range (BR) Spectra (Fermentas).

Viral protein expression in transfected 293T cells (passage 0) and Vero cells infected with recovered rRaV or rRaV/Xh viruses (passages 1 and 3) was investigated by Western blotting. Genome-length viral RNA transcripts generated using *in vitro* transcription from pT7-RaV and pT7-RaV/Xh plasmids and further post-transcriptional capping, led to the detection of mature NS3 (helicase) and VP1 (major capsid protein) synthesis (Figure 5A) during the virus passages studied, except in passage 0 where only a weak band corresponding to NS3 could be visualized. No viral proteins were detected using uncapped genome-length synthetic RNA transcripts, highlighting the key role of the 5′-cap structure in virus recovery. 293T cells infected with rFPV-T7 and further transfected with the infectious plasmids (passage 0) as well as Vero cells subsequently inoculated with the supernatants from 293T infected/transfected cultures were also studied for viral protein expression up to three passages. NS3 and VP1 were readily detected in samples from passages 1–3 (passage 2 not shown), although only NS3 could be detected in passage 0 (Figure 5B).

**FIGURE 6:**
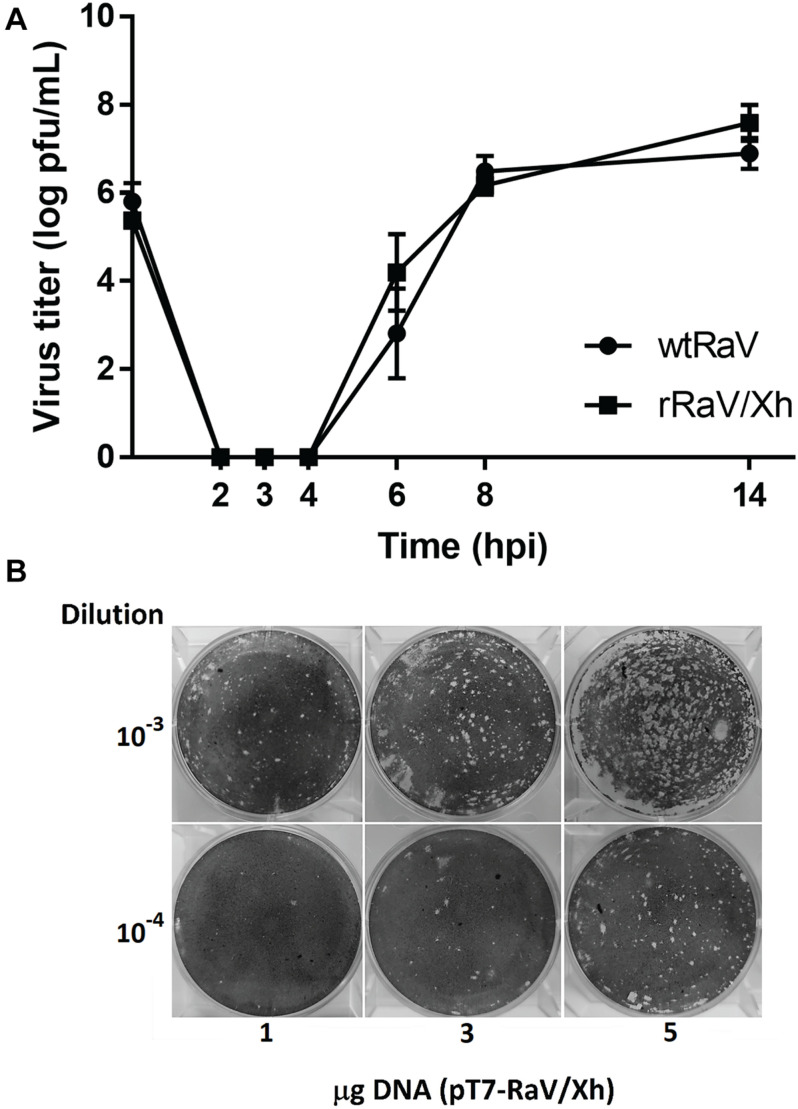
**(A)** One-step growth curves of wtRaV and rRaV/Xh in Vero cells infected at MOI = 10 and incubated for 14 h. No significant statistical differences were found between both viral titers within each time point (Student’s *t*-tests, *p* > 0.05). **(B)** Effect of the DNA amount used in transfection (passage 0) over the titer of viruses recovered in passage 1 supernatants. Transfections were performed in 6-well plates with either 1, 3, or 5 μg of the infectious cDNA clone. For each DNA quantity used in passage 0, plaque assay wells inoculated with the corresponding passage 1 supernatant dilutions 10^–3^ and 10^–4^ are shown.

To determine whether the rescued virus rRaV/Xh has growth features similar to those of wtRaV one-step growth curves were obtained after inoculation with each virus at MOI = 10. The results indicate that the growth phenotype of rRaV/Xh was similar to that of wtRaV (Figure 6A). Additionally, we wanted to investigate whether the initial amount of transfecting infectious clone has any effect over the rescued virus yield. For this purpose, we transfected different amounts of pT7-RaV/Xh plasmid after rFPV-T7 infection and inoculated the passage 0 supernatants into Vero cells. Following incubation, the viruses recovered from passage 1 were titrated. As shown in Figure 6B, the virus titer increased with the initially transfected DNA amount. After transfections with 1, 3 and 5 μg of pT7-RaV/Xh, the titers of recovered rRaV/Xh from passage 1 were 2 × 10^5^, 1 × 10^6^, and 1.08 × 10^7^ pfu/mL, respectively.

## Discussion

The establishment of reverse genetics systems for caliciviruses has been significantly delayed with respect to other RNA viruses due to the absence of efficient cell culture systems for most of these viruses, especially for those that infect humans, such as human noroviruses. Because of this, a few cultivable animal caliciviruses (e.g., feline calicivirus, murine noroviruses) have been used as surrogate model systems from which to extrapolate, infer and generalize aspects of the biology of this virus family (Goodfellow, 2013).

Due to the ability of RaV to efficiently propagate in a wide range of animal and human cell lines (Martín-Alonso et al., 2005), the establishment of a reverse genetics system for this virus has been one of our main objectives in recent years. Obtaining a full-length transcript from a cDNA clone of the virus genome is essential, but it does not guarantee its biological activity. Prior to this work, we were able to rescue a tagged RaV progeny at least once from a T7-driven genome-expressing vector named pT-A23/Xh (unpublished observation). All further attempts to reproduce this result were unsuccessful. However, this result was encouraging as it proved that a reverse genetics system for RaV could be feasible if we could find and clone the right viral genomic sequence. It has been reported previously that the lack of proofreading activity of RNA dependent RNA-polymerases found in RNA viruses makes them highly error prone so that many defective copies of the viral genome might be generated which contain single or multiple mutations that render them replication-incompetent (Boyer and Haenni, 1994). Normally, these defective genome copies would be eliminated in the following round of replication but, during the construction of infectious clones, the probability of amplifying, cloning and selecting a defective genome might not be low (Boyer and Haenni, 1994).

The main drawback of pT-A23/Xh plasmid was its inability to produce authentic viral RNA ends upon transcription: due to the cloning strategy used, the RNA derived from this vector has a non-viral 61 nt sequence upstream of 5′ virus genome end, and the lack of a proper transcription termination signal leads to a variable number of non-viral 3′ nucleotides added downstream of the poly-A tract. This is why we focused our work in constructing new RaV cDNA clones giving special emphasis to the generation of RNA transcripts that mimic, as closely as possible, what is thought to be the viral genome sequence, avoiding the incorporation of non-viral nucleotides at the 5′ and 3′ ends (Figure 1B).

This work reports the design and construction of vectors encoding the RaV genome under control of T7 bacteriophage RNA polymerase promoter. The genome-size synthetic RNAs generated were transfected into 293T cells, allowing the recovery of infectious virus progenies. In a different approach, in order to bypass the *in vitro* transcription step, virus recovery was also made by transfection of plasmid DNA into mammalian cell lines previously infected with rFPV-T7 (a recombinant poxvirus encoding T7 RNA polymerase). Both virus rescue systems have pros and cons: while synthetic transcript transfections have the advantage of eliminating the requirement for a T7 polymerase-expressing helper virus, it demands a higher level of care during handling of the labile RNA molecule and introduces the need for an RNase-free environment. Conversely, cDNA is more easily handled and less prone to degradation, but the required helper virus may interfere with the rescued virus replication, masking its CPE and may be toxic for the cells, thus has to be removed from the culture.

The appearance of virus-induced CPE and the detection of *de novo* synthesized viral RNA and proteins after three or more passages of culture supernatants demonstrated the recovery of infectious RaV progenies using both approaches. Both, 293T and Vero cells are permissive for RaV replication. 293T cells were selected for transfection experiments based on their well-known higher transfection efficiency. However, the virus-induced CPE was very difficult to observe in the transfected cells (passage 0), likely due to the cytotoxicity of the transfection reagent. In the case of DNA transfections, it was not possible to detect RaV-induced CPE, as it was masked by the CPE associated to fowlpox virus infection (Figure 2A). These fowlpox-associated damages included the loss of monolayer integrity, the appearance of rounded and refringent cells, as well as dead cells floating on the culture medium. In contrast, Vero cells shows very clear and easy-to-distinguish RaV-induced CPE. For this reason, this cell line was selected to test the efficacy of virus rescue: RaV-induced CPE was evident at passage 1, 24–72 h after inoculation of fresh Vero cells with the filtered supernatants from transfected 293T cultures (Figure 2C).

In the case of transfections using synthetic RNA, we observed clear differences between the morphology of cells transfected with capped RNA (seemingly positive for CPE) with respect to those transfected with uncapped RNA. As expected, the cells transfected with uncapped RNA were morphologically indistinguishable from the lipofectamine mock-transfected control (Figure 2B). This finding agrees with the lack of virus-induced CPE in subsequent supernatant blind passages and the impossibility to detect *de novo* synthesized viral RNA (Figure 4A) and proteins (Figure 5A) in these transfected cultures. These results suggest an essential role for the 5′-cap structure during RNA-based reverse genetics, as a substitute for the naturally occurring covalently linked VPg. The genome-linked protein is essential for calicivirus translation, as it is needed to recruit eukaryotic translation initiation factors and ribosomes during protein synthesis (Goodfellow et al., 2005; Chaudhry et al., 2006; Daughenbaugh et al., 2006). To date, because of the lack of an efficient method to covalently attach VPg to viral RNA synthesized *in vitro*, several calicivirus reverse genetics systems have been set up in which VPg was substituted by 5′-cap structures. Such replacement did not hamper the infectivity of rescued viruses, and the genomic RNA extracted from progeny virions had recovered the covalently linked VPg in its 5′ terminus, as it is encoded in the viral genome (Goodfellow, 2013).

Both the fowlpox virus-mediated *in vivo* RNA 5′-capping as well as the 5′-cap added post-transcriptionally using the ScriptCap m7G capping system, allowed the recovery of replication-competent RaV, in a reproducible manner. In this sense, our results agreed with previous studies suggesting a requirement for 5′-cap for the rescue of calicivirus from synthetic RNA transcripts (Sosnovtsev and Green, 1995; Chaudhry et al., 2007; Arias et al., 2012).

In an attempt to further characterize the recovered viruses, and to compare their phenotypes with that of wild type RaV, we analyzed the integrity and sequence accuracy of 5′ ends (Figure 3B) as well as the sequence of the full genome. While the sequence of the 5′ most terminal 430 nucleotides was conserved and identical to that of wtRaV, different degrees of nucleotide changes were recorded elsewhere throughout the genome, as it should be expected according to the error rate of viral RNA polymerase. Interestingly, most of the nucleotide changes recorded were transitions and most were silent (data not shown).

As for the detection of virus translation products, the non-structural protein NS3 (helicase) could be detected in the transfected monolayers (passage 0), as a faint protein band of the expected size, while the major structural protein VP1 was only detectable after several rounds of viral replication in subsequent virus passages (Figure 5). This could be explained because NS3 is a product from the proteolytic processing of polyprotein encoded in ORF1, which is produced immediately upon transfection in most transfected cells. On the other hand, VP1 is detected at later times because it is translated from the sgRNA, which requires the previous replication of RNA giving rise to genome-length negative strand from which sgRNA is made.

Based on our experience attempting to construct a RaV infectious clone, we hypothesize that the efficiency of virus rescue is directly related to the stability of genome-length transcripts. RNA must remain intact long enough for translation and replication to occur while in the presence of cytoplasmic cell nucleases (Wilusz et al., 2001; Dreyfus and Regnier, 2002). In addition to 5′-end protection against 5′ exoribonucleases conferred by the 5′-cap, the viral full-length transcripts required a 3′ end poly-A tail long enough to guarantee a minimal half-life time, allowing the genome to be copied by the replicase before 3′-exoribonucleases could reach the essential 3′-UTR region. In the past, we failed to reproducibly recover RaV using plasmids expressing RaV genomic cDNA followed by a poly-A tail with a fixed length of 15 nt (unpublished data). Several successful reverse genetics systems for caliciviruses included poly-A tails of 30 nt or more (Chang et al., 2005; Yunus et al., 2010). In the work presented here, we doubled the length of the poly-A tract in the RaV genetic constructs (up to 30 nt) and we believe this might be one of the reasons (though perhaps not the only one) behind the successful rescues. We speculate that further increasing the length of poly-A tract could improve the rescue efficiency and increase the titer of recovered virus, though this needs further investigation.

The one-step growth curve for the tagged recovered virus rRaV/Xh was very similar to that obtained for wtRaV, with a prominent eclipse phase between 2 and 4 hpi during which no viral lysis plaques were recorded (Figure 6A). This similarity among growth curves suggest that the *Xho*I mutation introduced as a molecular tag in the infectious clone has no deleterious effect on RaV phenotype. The infectious titer of recovered viruses after passage 1 increased with the initial pT7-RaV/Xh plasmid amount used in transfection in a concentration-dependent manner (Figure 6B). This finding represents an opportunity for further optimization of this reverse genetics system, based on transfection set up. For example, higher titers of rescued virus could be achieved in shorter periods of time by increasing the amount of transfected DNA.

Because of the ability of *Rabbit vesivirus* to easily infect and propagate to high titers in many cell lines derived from different host species, including humans, as well as the well-known ability of some vesiviruses to cross the species barrier, special care should be taken handling RaV as it could become a potential emerging pathogen then deserving full attention. This work is relevant as far as it provides a powerful molecular biology tool to genetically manipulate this viral pathogen to study viral gene functions and their role in pathogenesis. The RaV infectious clone will also be an invaluable tool for our lab to interrogate this virus’ capacity to tolerate foreign gene insertions, viral gene replacements, as well as its performance as a potential vaccine candidate against unrelated animal or human viral infections.

## Data Availability Statement

The datasets presented in this study can be found in online repositories. The names of the repository/repositories and accession number(s) can be found below: https://www.ncbi.nlm.nih.gov/genbank/, MN179279.

## Author Contributions

KD, JM-A, and FP conceptualized the research and provided supervision. AA, AG-M, IN, AP, MA-Z, and DL performed the research. AA and AG-M analyzed the data. FP was involved in funding acquisition. AA wrote the manuscript. All authors contributed to the article and approved the submitted version.

## Conflict of Interest

The authors declare that the research was conducted in the absence of any commercial or financial relationships that could be construed as a potential conflict of interest.
